# An Outbreak of Fearsome Photos and Headlines: Ebola and Local Newspapers in West Africa

**DOI:** 10.4269/ajtmh.16-0245

**Published:** 2016-11-02

**Authors:** Eric S. Halsey

**Affiliations:** 1 The President's Malaria Initiative, Atlanta, Georgia; 2 Malaria Branch, Division of Parasitic Diseases and Malaria, Centers for Disease Control and Prevention, Atlanta, Georgia

“Let me get this right, the pilot wants to talk with me in the cockpit?” I asked the flight attendant, as the final passengers boarded the Liberia-bound jet during a refueling stop in west Africa. It was late March 2014, and a small Ebola virus disease (EVD) outbreak had been reported a few weeks earlier in Guinea. The half-read pile of malaria journal articles on my lap provided the flight crew sufficient evidence to conclude there was a “tropical medicine guy” on the flight, just what they needed for their current dilemma. As I walked toward the front of the plane, I had a suspicion what the upcoming topic of discussion would be.

“When I told company headquarters I was not flying this plane to Monrovia, they threatened to fire me,” the pilot anxiously greeted me and then added, “You know the first case of Ebola in Liberia was diagnosed a few hours ago. I can have us headed back to the U.S., especially if you agree. Doc, what do you think, should we really be flying to Liberia right now?”

With important malaria-related meetings awaiting me in Monrovia and an understanding of the minimal risk a single, unconfirmed case of EVD posed to the pilot and crew, I replied, “I think you have nothing to worry about” and went on to explain the fundamentals (of what was known at the time) of Ebola virus transmission. Our plane was Liberia bound a few minutes later and, although I did not realize it at the time, that would be the first of many instances of Fearbola I would encounter in west Africa.

In public health, one of the first rules of managing an outbreak is to communicate early, truthfully, and effectively. For public health workers, this involves conveying appropriate information related to disease manifestations and prevention strategies. During such a time, the press is also in the business of conveying information, but may do so with a different set of principles and objectives.

In early Autumn 2014, the word “Fearbola” was popularized as a description of the public's reaction to the intensity and nature of the initial press coverage of a handful of EVD cases in the United States.^1^,^2^ Airline flights were cancelled, students were denied admission to school, and many with negligible risk were quarantined. Similar to how it dominated U.S. news reports, EVD also became a recurring front-page story in west African newspapers during the epidemic which has now killed more than 11,000 people in Guinea, Liberia, and Sierra Leone.^3^ By perusing local newspapers during the first 2 weeks of the EVD epidemic in Liberia (with which my visit just happened to coincide)—and a year later during an EVD deployment in Sierra Leone—I learned that Ebola sensationalism was not the exclusive domain of Westerners or their media.

Throughout sub-Saharan Africa, newspapers remain an important method of communication, with the trend of independent newspapers crowding out government-funded and controlled ones.^4^ These privately owned, independent newspapers may enjoy a less restrictive atmosphere than their political-mouthpiece counterparts, but often face challenges in establishing a circulation or advertising base sufficient to cover operating expenses. Consequently, some newspapers may resort to “gombo journalism” (where reporters receive money for covering a story) or “beat associations” (where an identical story appears in numerous newspapers under a different title), whereas others may favor, to use one observer's description, the “trivialization and tabloidization of the news” to garner more subscription or advertising revenue.^4^

In late March and early April 2014, immediately after the announcement of the first case of EVD in Liberia, local newspapers displayed titillating front-page messages: “Ebola Terror Rages” (Figure 1

**Figure 1.**
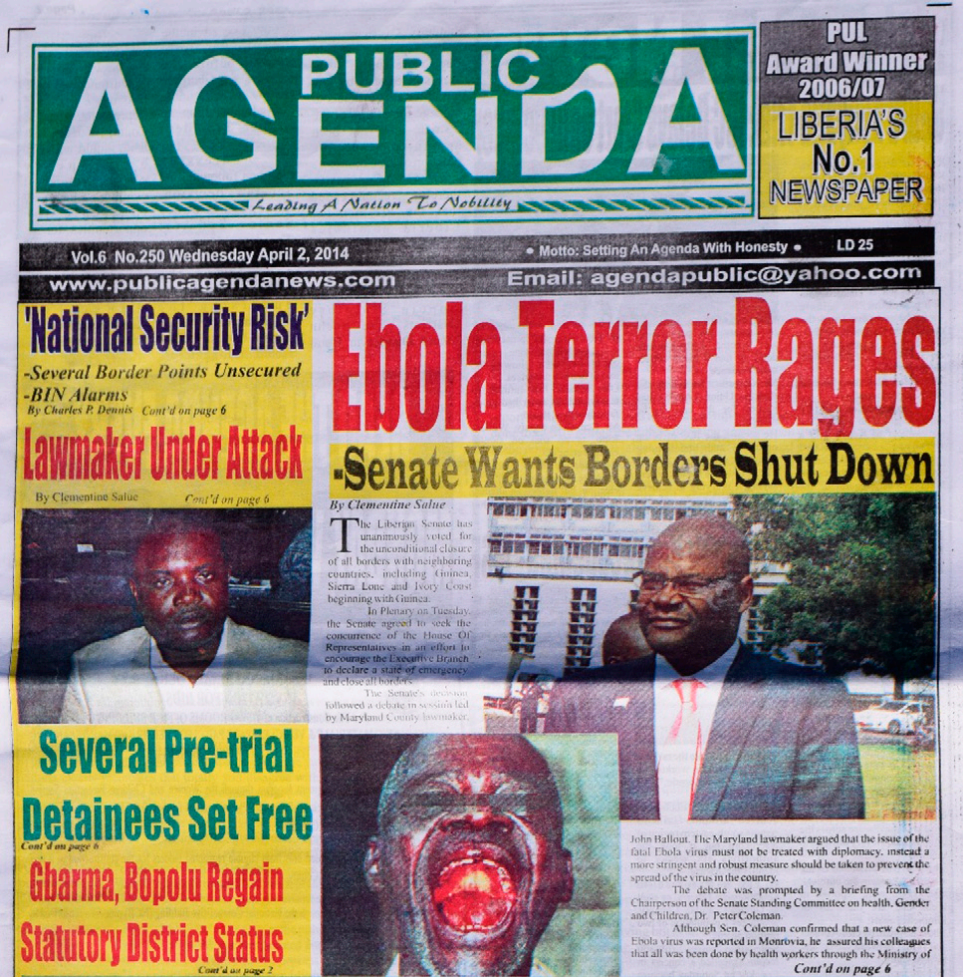
Public Agenda (Liberian newspaper), April 2, 2014.

) and “State of Emergency Looms As Ebola Fear Reaches New Heights.” Others, undaunted by an inability to obtain authentic photographs to accompany sensationalist headlines, borrowed images from the Internet. One image—likely from a horror movie—displayed a man clad in a bow tie, clutching his face as bloody eyeballs bulged from his sockets with the headline, “Ebola Claims Ten Lives.” (Figure 2

**Figure 2.**
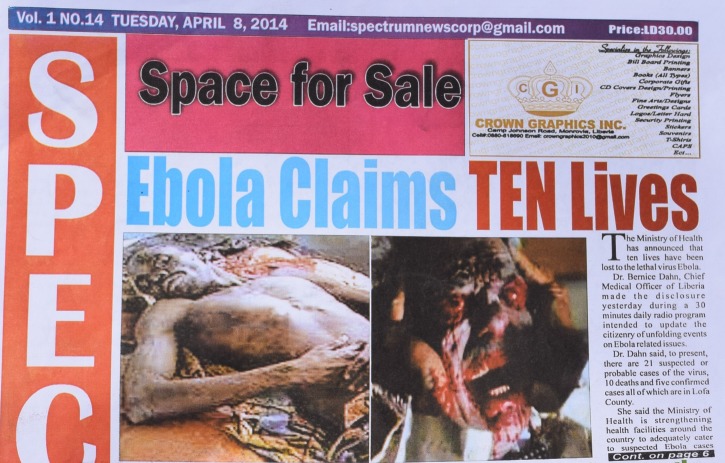
Spectrum News (Liberian newspaper), April 8, 2014.

). Another newspaper showed blood trickling down a heavily pock-marked face of what appears to be a mannequin (Figure 3

**Figure 3.**
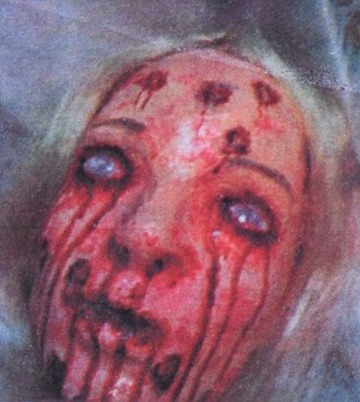
Women Voices (Liberian newspaper), April 7, 2014.

).

In addition, faulty public health messages were propagated from some periodicals, such as a front-page picture of a produce stand with the sign, “Please Use Gloves Before Touching Vegetables and Fruits” (Figure 4

**Figure 4.**
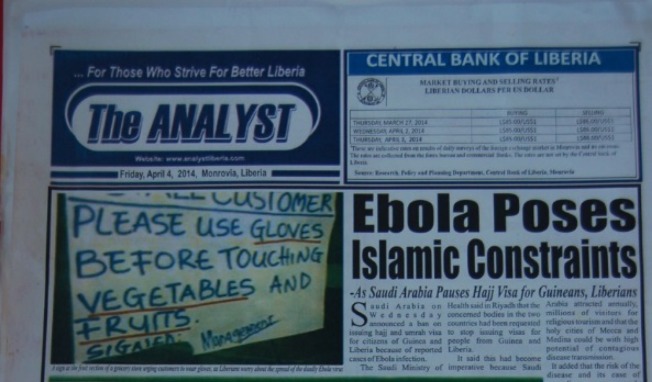
The Analyst (Liberian newspaper), April 4, 2014.

). Another errantly exclaimed: “More Fatalistic than AIDS, Kills in Seconds! MOH [Ministry of Health] Confirms Ebola in Liberia” (Figure 5

**Figure 5.**
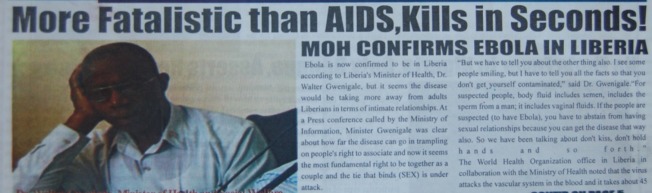
The West African Pilot (Liberian newspaper), April 3, 2014.

).

Editorialists exploited the situation to convey reproachful religious messages. One declared, “Ebola virus which is almost becoming a national plague…clearly signals the need for caution to Liberians to lead lives that will avoid the wrath of God descending on this nation through plagues like Ebola and judgment like that of the twin cities of Sodom and Gomorrah.” Another article titled, “Liberians need Jesus to combat Ebola,” featured the quotes of a local educator placing blame on the country's sinners and their “total disregard for the Creator.”

Such embellishments were not limited just to the initial weeks of the epidemic or to Liberia. In October 2014, one Sierra Leonean paper proclaimed, “Ebola victim rises from the dead,” alongside a dubious photograph probably borrowed from Hollywood (Figure 6

**Figure 6.**
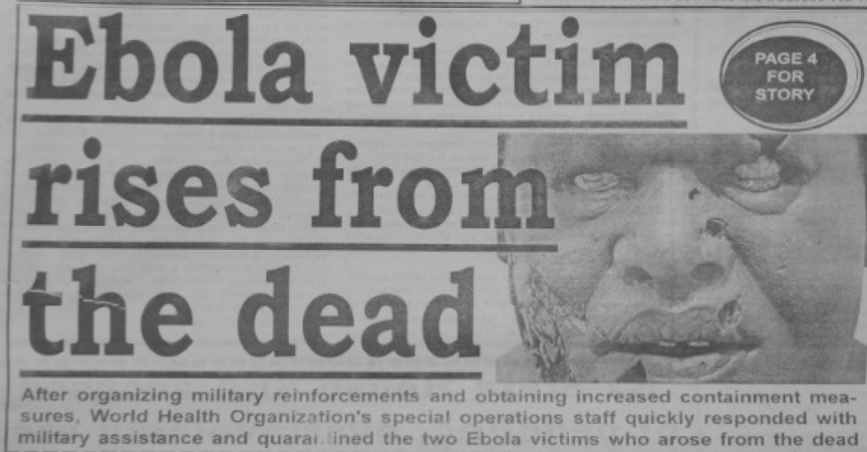
Independent Observer (Sierra Leonean newspaper), October 10, 2014.

). In January 2015, a different Sierra Leonean newspaper featured a story with the headline, “Blood of Survivors Cures 35 out of 40” (Figure 7

**Figure 7.**
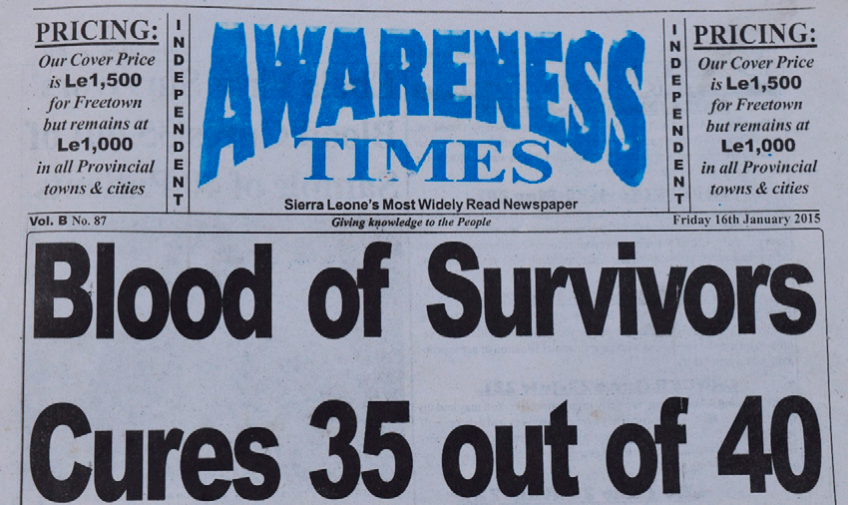
Awareness Times (Sierra Leonean newspaper), January 16, 2015.

).

A misinformed, paranoia-driven community may pose danger beyond just disease transmission. In April 2014, EVD control efforts were jeopardized in Macenta, Guinea, when a group attacked a treatment site, alleging that foreign health-care workers had brought the disease.^5^ Similar fears sparked a frenzy in Womey, Guinea, in September 2014, when a mob killed eight outsiders visiting during an EVD education mission.^6^ Panic-stricken crowds destroyed a treatment center in Liberia in August 2014 and stoned contact tracers in Sierra Leone in May 2014.^7^,^8^ Although it is unlikely these incidents were directly caused by an individual sensational news story, the faulty messages promulgated by some local press outlets almost certainly added to the climate of fear and uncertainty.

But the press can be used in positive ways and, to be fair, much of the local coverage in west Africa was responsible and accurate. Within a week of the country's first case, one Liberian newspaper page titled, “Fighting Against Ebola We Are Together,” incorporated the Ministry of Health and Social Welfare's messages about modes of disease transmission and provided prevention strategies, including what to do after contact with a potential case and a number to call for those with questions (Figure 8

**Figure 8.**
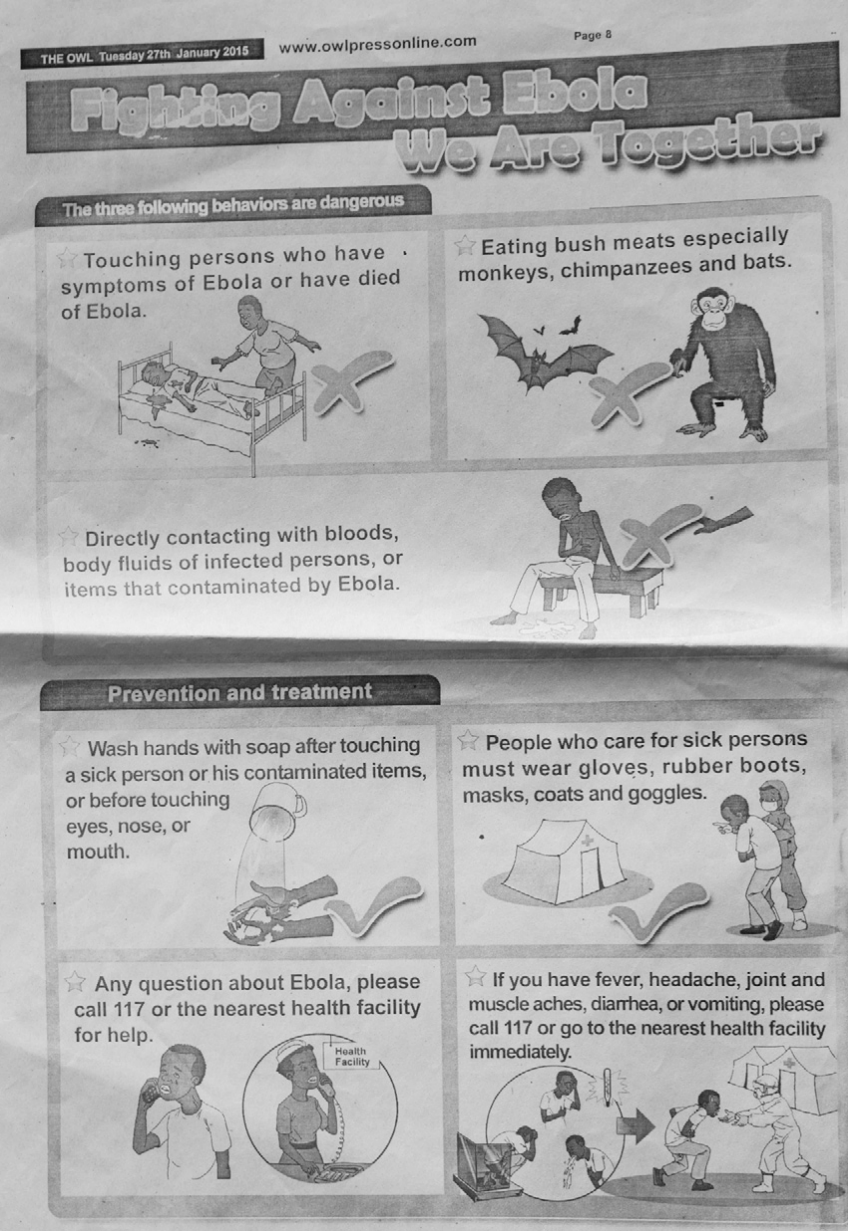
The Owl (Sierra Leonean newspaper), January 27, 2015.

). James Harding Giahyue, editor-in-chief of the Liberian newspaper, The Catalyst, during the EVD epidemic, described his initial concerns: “For me, I thought I could not be sensational about Ebola because I thought it came with its own notoriety—a kind of touch-and-catch disease—for which it would have been redundant to exclaim…I knew Ebola was more than a security issue. It was something that required an editor's meticulous judgment.”

International groups were also able to partner with the media. During the height of the epidemic, nongovernmental organizations, such as Internews, offered workshops to Liberian journalists who, in turn, used their platforms to educate about EVD. One campaign, which featured the slogan “Ebola Must Go,” was championed by the country's president, Ellen Johnson Sirleaf, and spanned multiple media formats beyond just newspapers, including music videos, radio jingles, billboards, Facebook, and Twitter. Agencies like the U.S. Centers for Disease Control and Prevention worked alongside the Ministry of Information, Cultural Affairs and Tourism of Liberia to ensure scientifically valid information was contained in daily press briefings.

Since the waning of the EVD epidemic, efforts remain to provide sub-Saharan journalists skills to better cover scientific stories of public interest.^9^ In late 2015 and early 2016, the World Federation of Science Journalists conducted a series of 5-day training workshops in Liberia, Guinea, and Sierra Leone. In conjunction with these sessions, information was collected from participants about journalistic challenges faced during the epidemic. This information will be analyzed and used to inform future workshops, targeting reporters from other countries in the region and focusing on barriers and best conditions needed to enhance information uptake and capacity building in health and science communication. Appropriate media communication strategies conveying accurate and timely information cannot, of course, prevent every outlandish story or bizarre photograph, but they can be an important first step in advocating for behavioral change to limit disease transmission when the inevitable next outbreak surfaces.

## References

[1] James JJ (2014). Fearbola. Disaster Med Public Health Prep.

[2] Towers S, Afzal S, Bernal G, Bliss N, Brown S, Espinoza B, Jackson J, Judson-Garcia J, Khan M, Lin M, Mamada R, Moreno VM, Nazari F, Okuneye K, Ross ML, Rodriguez C, Medlock J, Ebert D, Castillo-Chavez C (2015). Mass media and the contagion of fear: the case of Ebola in America. PLoS One.

[3] Centers for Disease Control and Prevention (2016). 2014 Ebola Outbreak in West Africa: Case Counts.

[4] Gicheru C (2014). The Challenges Facing Independent Newspapers in Sub-Saharan Africa.

[5] Thomson Reuters (2014). Mob Attacks Ebola Treatment Centre in Guinea, Suspected Cases Reach Mali.

[6] CTV NEWS (2014). Mob in Guinea Kills 8 Officials, Journalists on Ebola Awareness Trip.

[7] PBS NEWSHOUR (2014). Liberian Mob Attacks Ebola Clinic; Dozens of Patients Missing.

[8] Patric Foryoh Report (2014). Villagers Stone Workers Tracking Ebola in Sierra Leone.

[9] Chalaud D, Aghan D, Otindo V, Bennett A, Baldet T (2015). Ebola: improving science-based communication and local journalism. Lancet.

